# The Influence of Modifiable Factors on Breast and Prostate Cancer Risk and Disease Progression

**DOI:** 10.3389/fphys.2022.840826

**Published:** 2022-03-07

**Authors:** Keely Tan, Matthew J. Naylor

**Affiliations:** Charles Perkins Centre, School of Medical Sciences, Faculty of Medicine and Health, University of Sydney, Sydney, NSW, Australia

**Keywords:** breast cancer, prostate cancer, modifiable risk factors, lifestyle, environmental, inflammation, reactive oxygen species

## Abstract

Breast and prostate cancers are among the most commonly diagnosed cancers worldwide, and together represented almost 20% of all new cancer diagnoses in 2020. For both cancers, the primary treatment options are surgical resection and sex hormone deprivation therapy, highlighting the initial dependence of these malignancies on the activity of both endogenous and exogenous hormones. Cancer cell phenotype and patient prognosis is not only determined by the collection of specific gene mutations, but through the interaction and influence of a wide range of different local and systemic components. While genetic risk factors that contribute to the development of these cancers are well understood, increasing epidemiological evidence link modifiable lifestyle factors such as physical exercise, diet and weight management, to drivers of disease progression such as inflammation, transcriptional activity, and altered biochemical signaling pathways. As a result of this significant impact, it is estimated that up to 50% of cancer cases in developed countries could be prevented with changes to lifestyle and environmental factors. While epidemiological studies of modifiable risk factors and research of the biological mechanisms exist mostly independently, this review will discuss how advances in our understanding of the metabolic, protein and transcriptional pathways altered by modifiable lifestyle factors impact cancer cell physiology to influence breast and prostate cancer risk and prognosis.

## Introduction

**Figure 1:**
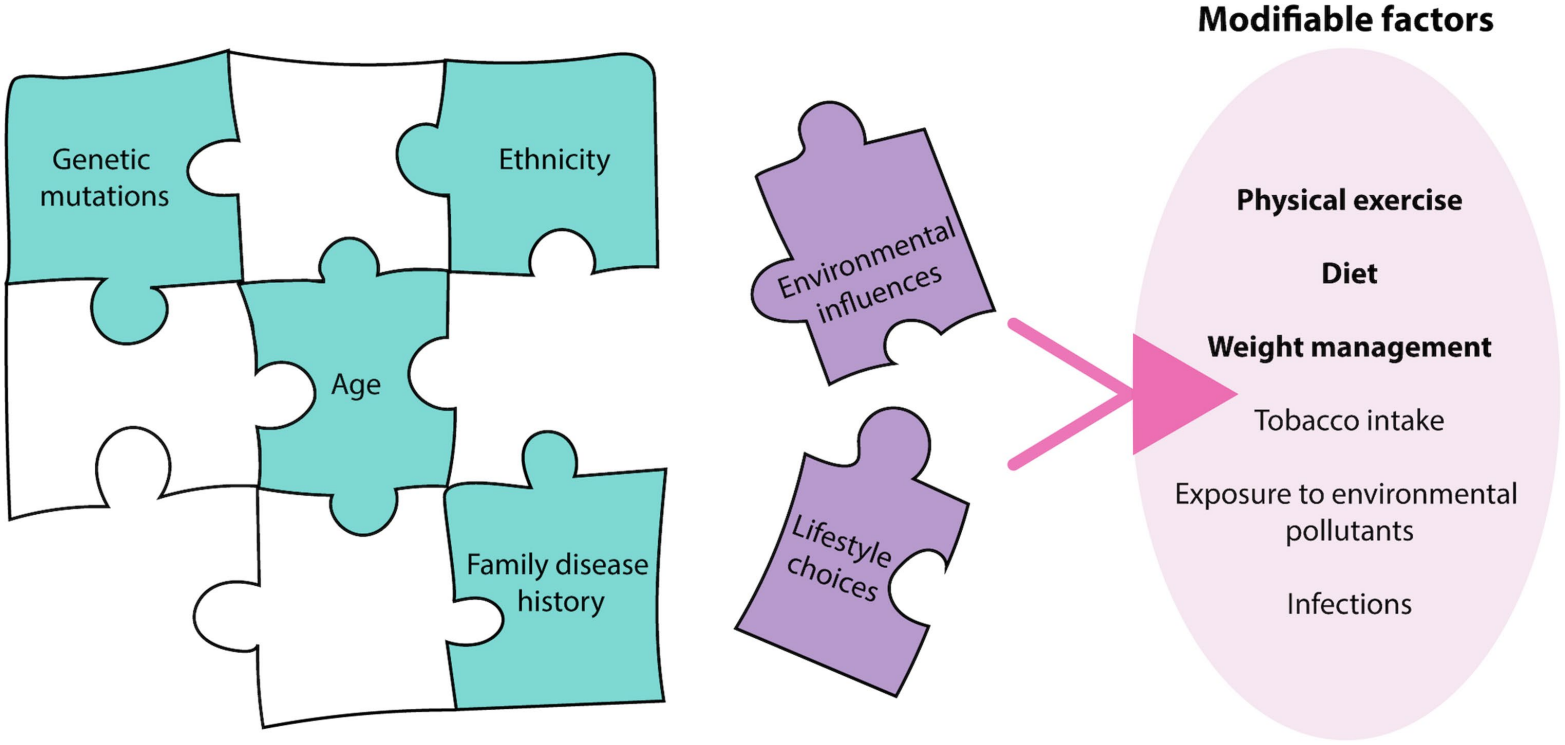
Breast and prostate cancer etiology. The etiology of breast and prostate cancer relies on many pieces of a complex puzzle, where environmental influences and lifestyle choices, termed modifiable factors, may complete this puzzle. There are various modifiable factors that may contribute to cancer initiation, with physical exercise, diet, and weight management most relevant to breast and prostate cancer.

Breast and prostate cancers are among the most commonly diagnosed cancers worldwide, representing 19% of all new cancer diagnoses and 10.7% of cancer-related deaths in 2020 alone (Sung et al., 2021). The pathophysiology of these cancers relies on the complex interplay and exploitation of various biological systems, with systems biology techniques, such as ‘omic’ approaches, now being employed to understand their pathogenesis (Du and Elemento, 2015). Past research in breast and prostate cancer predominantly focused on aberrations in the human genome driving disease development, but it is now increasingly apparent that this represents only one piece of the complex cancer puzzle (Wang et al., 2018; Wu et al., 2018). While genome and other non-modifiable factors such as age, ethnicity and family history contribute to an individual’s disease risk (Nindrea et al., 2017), factors associated with lifestyle choices and environmental influences are becoming increasingly recognized as additional pieces that complete this puzzle (Stein and Colditz, 2004) (Figure 1). Breast and prostate cancer risk of immigrants originally from low disease prevalence countries, increases to reflect that of the destination country (Shimizu et al., 1991; Kolonel et al., 2004), confirming the importance of external factors in the etiology of the diseases. A study of twins demonstrated that heritable factors contributed to 42 and 27% of an individual’s risk for prostate and breast cancer, respectively, (Lichtenstein et al., 2000), further demonstrating the major contribution of external factors to disease risk. In addition to being significant risk factors, external factors also influence disease progression post-diagnosis (Davies et al., 2011; Cannioto et al., 2021). As a result, lifestyle changes are being encouraged by health professionals as strategies for cancer prevention, and are thought to have the potential to prevent up to 50% of all cancer cases in developed countries (Stein and Colditz, 2004). Despite current population data demonstrating the significant impact of these modifiable factors on disease progression, the mechanisms of how these external factors influence cell biology to impact cancer phenotype and disease progression is not well understood. This mini review will describe how these modifiable factors can affect cellular systems, including the epigenome, transcriptome, proteome and cellular metabolome, which ultimately determines cancer phenotype.

## Epidemiological Evidence

**Figure 2:**
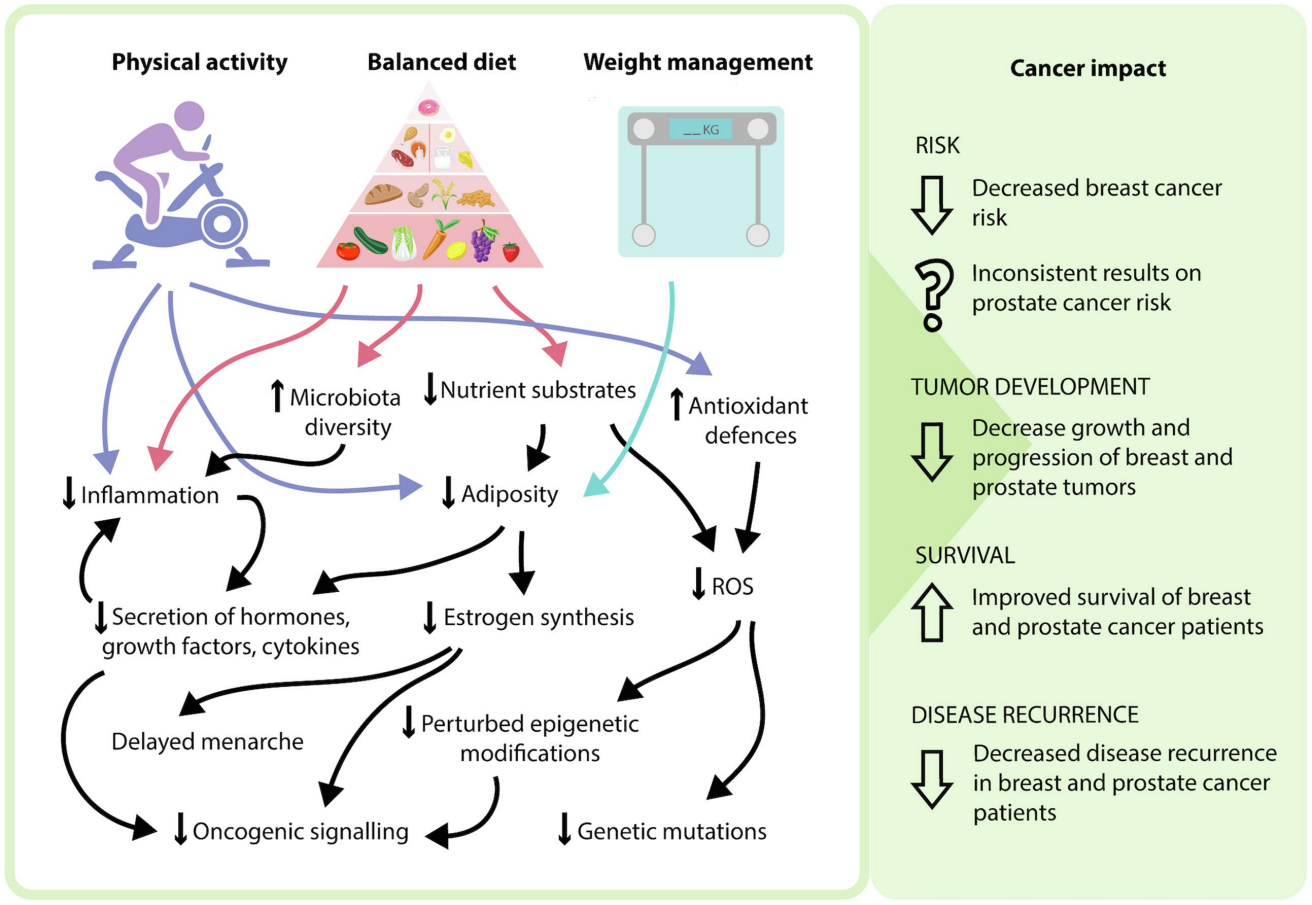
Potential mechanisms of how modifying lifestyle factors can influence cancer phenotype. In general, a side effect of increased physical activity and a balanced diet is weight management and adipose tissue loss. The incorporation of these three modifiable lifestyle factors can result in various physiological effects, including a decrease in nutrient substrates, adipose tissue, proinflammatory processes, reactive oxygen species-mediated effects, and oncogenic signaling, as well as an increase in antioxidant defenses and microbiota diversity. At the patient level, this may explain the reduced risk of breast cancer, decreased progression of breast and prostate cancers, as well as increased survival and decreased disease recurrence that occurs with modifying these lifestyle factors.

Modifiable risk factors encompasses both lifestyle choices and environmental exposures. These include physical exercise, diet, weight management, tobacco intake, exposure to environmental pollutants and infections (Stein and Colditz, 2004). These factors can contribute to an individual’s disease risk, recovery rate and likelihood of disease recurrence, with physical exercise, diet, and weight management being most relevant to breast and prostate cancer (Figure 1). Current epidemiological evidence highlights the positive effects of increasing physical exercise, a healthy diet and maintaining a healthy weight in the prevention and overall disease outcomes for breast cancer patients (Cannioto et al., 2021; Lubian Lopez et al., 2021). Interestingly, the impact of lifestyle interventions on prostate cancer risk has been inconsistent, with some studies demonstrating no effect, while others show decreased disease risk (Shephard, 2017; Sorial et al., 2019). While the contribution to disease risk is controversial, the consensus is that these interventions are beneficial in decreasing an individual’s risk of mortality and improving overall outcomes (Kenfield et al., 2011). As a result, it is important to understand the mechanisms of how these modifiable factors can influence patient risk and disease progression to effectively implement these strategies in the clinic. The physiology behind the lifestyle interventions resulting in these outcomes is complex, multi-factorial and often overlap with one another. Implementation of these lifestyle factors may modulate the impact of certain biological molecules, combat the chronic inflammatory state of tumors, decrease the expression and activity of pro-oncogenic genes and signaling pathways through epigenetic mechanisms, and improve regulation of oxidative stress to minimize oxidative damage (Figure 2).

## Metabolic and Hormonal Influence

The response to lifestyle and environmental cues occurs initially at the metabolic and hormonal level, which can dynamically alter gene expression through epigenetic and transcriptional mechanisms (Wong et al., 2017). While food consumption stimulates the release of hormones and metabolites such as insulin and insulin-like growth factor (IGF)-1 (Clemmons, 2012; Vernieri et al., 2016), overnutrition is linked to the perturbed activity of these hormones. The increased activity of insulin and IGF-1 result in the activation of oncogenic signaling pathways and subsequently increase proliferation and disease progression (Pollak, 2012). In addition, metabolic substrates derived from lipids, protein and carbohydrates can provide a constant supply of ATP and metabolic precursors for biochemical processes crucial for tumor progression, such as lipid membrane synthesis (Hanahan and Weinberg, 2011; Vernieri et al., 2016). Furthermore, there is a plethora of evidence to support the link between nutritional choices and gut microbiota composition, with low microbiota diversity associated with cancer (Plaza-Diaz et al., 2019; Wastyk et al., 2021). Multi-omic approaches have been used to link gut microbial dysbiosis with the advancement of breast and prostate cancer (Komorowski and Pezo, 2020; Liu et al., 2021). Evidence indicates that this may be due to the contribution of dysbiosis-related metabolites in chronic inflammation, immune cell recruitment and cancer cell dissemination (Buchta Rosean et al., 2019; Lee et al., 2021). Using metagenomics, Liu and colleagues demonstrated that dysbiosis accelerated prostate cancer progression through upregulation of lysophosphatidylcholine acyltransferase 1 (LPCAT1), a key enzyme in the phospholipid remodeling pathway (Liu et al., 2021). In addition, the gut microbiome-associated metabolites may influence cancer progression indirectly by altering the breast microbiome through systemic effects (Costa et al., 2021). Extending upon this, nutritional metabolomics can provide detailed analyses of metabolites related to the consumption of certain foods, such as alcohol and animal fats, which can be predictive of breast and prostate cancer risk. For example, elevated lysophosphatidylcholines C17:0 and C18:0 levels have been associated with increased prostate cancer risk (Playdon et al., 2017; Röhnisch et al., 2020). As nutrition has the potential to contribute to tumor growth through the discussed metabolic mechanisms, dietary interventions such as “short-term fasting” have been trailed and found to reduce blood glycemia, hyperinsulinemia and IGF-1 levels (Vernieri et al., 2016). Furthermore, participation in physical activity can influence hormone and metabolite levels, such as decreasing insulin and IGF-1 levels, thus reducing their oncogenic effects (Thomas et al., 2017).

In addition to metabolic disruptions, ongoing overnutrition results in adipose tissue accumulation. Adipose tissue is known to be a source of estrogen production, particularly in postmenopausal women whose ovaries are no longer the major estrogen source (Hetemaki et al., 2021). Postmenopausal women with increased BMI or weight have an increased risk of developing hormone receptor positive breast cancer (Brown et al., 2017). Aromatase, a key enzyme involved in estrogen biosynthesis, is expressed in adipose tissue with increased BMI correlating with increased aromatase expression (Zhao et al., 2016). Estrogen has a demonstrated role in breast cancer initiation, proliferation and progression (Han et al., 2018; Xue et al., 2019), subsequently estrogen exposure has been strongly linked to the development of breast cancer, even in premenopausal women. In fact, there is a 5% increased risk of breast cancer correlated with each year younger at menarche and a 3.5% increase related to each year older at menopause due to the prolonged period of estrogen exposure (Ramakrishnan et al., 2002; Collaborative Group on Hormonal Factors in Breast, 2012). There are various factors that can promote the onset of menarche, with diet, physical activity and BMI being recognized as contributing external factors (Ramezani Tehrani et al., 2014). Diet has been closely linked to menarcheal age, with overnutrition and obesity correlated with decreased age, and undernutrition associated with an increased age to onset of menarche (Merzenich et al., 1993). While this correlation between diet, obesity and spermarche may also be evident in boys, the relationship is harder to determine given that it is more difficult to determine spermarche onset (Wagner et al., 2012; Deng et al., 2018). Research indicates that testosterone levels may not explain the potential relationship between obesity and spermarche, given that obesity has been associated with lower testosterone levels (Glass et al., 1977), however elevated leptin associated with increased adiposity has been highlighted as a potential mediator of pubertal age (Wagner et al., 2012). This may suggest that dietary choices as early as childhood, could contribute to an individual’s breast and prostate cancer risk later in life.

## Immune Function and Inflammation

Adipose tissue, a major consequence of an unhealthy diet and a sedentary lifestyle, is comprised of adipocytes, adipose stem cells, endothelial cells, immune cells and fibroblasts. Adipose tissue can secrete a range of hormones, growth factors and cytokines, termed adipokines (Gilbert and Slingerland, 2013; Lenz et al., 2020). The balance of these factors is dependent on the composition of the adipose tissue, with the onset of obesity identified as a driver of adipose remodeling. This alters the size and composition of adipose tissue, with an increase in preadipocytes and a decrease in mature adipocytes (Picon-Ruiz et al., 2017). The hypertrophy and proliferation of adipose tissue that occurs with progressive weight gain eventually results in adipose tissue hypoxia, triggering hypoxia-inducible factor-1 (HIF1) transcriptional activity (Lee et al., 2014). Recent multi-omic analysis has revealed that HIF1 transcriptional activity is dependent on its cofactor CDK8, which indirectly represses MYC target genes as an adaptive response to promote cell survival (Andrysik et al., 2021). In addition, HIF1 activity upregulates other genes, including vascular endothelial growth factor, which promotes angiogenesis and metastasis of breast and prostate cancer cells (Li et al., 2018; Melegh and Oltean, 2019). Increased HIF1 activity, and the predominantly preadipocyte phenotype, also increases leptin levels while decreasing adiponectin levels, propagating a proinflammatory environment (Gilbert and Slingerland, 2013). The imbalance of these hormones transforms the adipose tissue immune landscape, increasing the recruitment of various proinflammatory immune cells, such as macrophages, resulting in increased immune cell infiltration (Wu et al., 2019). These proinflammatory immune cells in conjunction with the preadipocytes, increase the secretion of inflammatory adipokines such as tumor necrosis factor alpha (TNF-α) and interleukin (IL)-1β, creating a chronic inflammatory condition associated with tumorigenesis (Gilbert and Slingerland, 2013; Picon-Ruiz et al., 2017). The preadipocyte phenotype discourages mature adipocyte differentiation, thus maintaining a proinflammatory state. However, this elevated immune cell mobilization and infiltration is not limited only to states of high adiposity and is typical of breast and prostate cancers (Wu et al., 2020; Xu et al., 2021). Thus, lifestyle interventions such as physical exercise, may improve the inflammatory state of all patients (Khosravi et al., 2019). While the exact mechanisms are not fully understood, one hypothesis is that exercise reduces monocyte cytokine production (Khosravi et al., 2021).

In addition to the impact of adipose tissue expansion through overnutrition, the uptake of certain nutrients, such as saturated fatty acids (SFAs), can also trigger inflammation. SFAs induce toll like receptor (TLR) activation, particularly TLR4 (Rogero and Calder, 2018), with activation of the TLR pathway resulting in increased activity of the transcription factor nuclear factor kappa-light-chain-enhancer of activated B cells (NF-kB), which is responsible for regulating over 100 proinflammatory genes (Pradere et al., 2014), further perpetuating a chronic inflammatory state. Therefore, nutrition choices and the accumulation of adipose tissue may influence the tumor microenvironment required for breast and prostate cancer growth and progression. By actively increasing levels of physical exercise and incorporating a heathier diet, this may decrease adipose-associated inflammation. In addition to the effects of decreased adipose tissue accumulation, partaking in physical exercise, particularly aerobic focused activity, has the capacity to improve immunity and reduce inflammation through the activation of β-adrenergic receptor (β-AR) signaling (Hong et al., 2014). Upon binding of circulating catecholamines to the β-AR of immune cells, adenylyl cyclase is activated to produce cAMP and activate PKA. The functional consequences of activating this pathway are dependent on the immune cell subtype (Simpson et al., 2021), but a hypothesized mechanism is that exercise-induced activation of the β-AR signaling pathway diminishes the TNF proinflammatory signaling axis, although this relationship is not as strong in obese individuals (Hong et al., 2014). Furthermore, physical exercise has been linked to alterations of the lipid profile and cytokine levels, such that there is an increase in high-density-lipoprotein levels and IL10 levels, respectively. Modulation of these parameters is associated with decreased chronic inflammation (Koelwyn et al., 2015; Meneses-Echavez et al., 2016). A recent study has also used multi-omic and immune profiling to demonstrate striking benefits of a high-fermented-food diet. This diet increased gut microbiome diversity, as well as decreasing inflammatory markers, such as IL-6 and IL-10 (Wastyk et al., 2021). While this study was only performed in healthy adults, there have been some *in vitro* and *in vivo* studies highlighting the benefits of fermented foods in breast and prostate cancer, but these findings are yet to be confirmed in the clinic (Tasdemir and Sanlier, 2020).

## Regulation of Oxidative Stress-Induced DNA Damage

It is well established that the role of reactive oxygen species (ROS) is paradoxical, in that it has the potential to be beneficial and detrimental to the progression of tumors, depending on the balance of antioxidants (Aggarwal et al., 2019; Perillo et al., 2020). For simplicity, this review will only discuss the pro-tumorigenic impact of ROS. This notion of oxidative stress arises from inefficient clearance of excess free radicals, and is commonly associated with the initiation of cancers, as it can cause oxidative damage to lipids, proteins and DNA, contributing to genomic instability and mutation (Sharifi-Rad et al., 2020). This process can occur naturally with aging, from external environmental stressors, such ultraviolet radiation, and also from lifestyle factors, such as nutritional choices. During overnutrition, the uptake of carbohydrates, lipids and protein trigger the production of ROS, predominantly due to the excess supply of energy substrates for mitochondrial metabolism (McMurray et al., 2016; Saha et al., 2017). This continued state of overnutrition can result in mitochondrial dysfunction and further increase oxidative stress and oxidative stress-induced DNA damage. In addition to food consumption, there has also been a strong link between alcohol intake and breast and prostate cancer risk through the production of ROS species and acetaldehyde arising from alcohol metabolism (Dickerman et al., 2016; Wang et al., 2017).

In a pre-malignant context, increased ROS levels provide the opportunity for driver somatic mutations to occur, which during malignancy can drive phenotypes such as cell proliferation (Perillo et al., 2020) and epithelial-mesenchymal transition (EMT) (Radisky et al., 2005) important for metastatic progression. In addition, multi-omics approaches have identified different cancers exhibit varied levels of ROS metabolism, and are beginning to investigate the use of a ROS index to measure cancer outcomes (Shen et al., 2020). Thus, the implementation of diet changes and weight management could influence the amount of oxidative stress and subsequently minimize the effects on cellular damage prior to and following the initiation of carcinogenesis. In addition to dietary modifications, research has indicated that participation in regular, and moderate to high-intensity physical exercise may improve antioxidant defenses both in adult and elderly individuals by upregulating antioxidant enzymes, allowing the body to adopt mechanisms to effectively process large quantities of ROS (Powers et al., 2020). These adaptive mechanisms may be beneficial in managing the potential increase in oxidative stress to decrease the risk and rate of mutation accumulation, and subsequent disease initiation. Conversely, the pro-tumorigenic role of ROS is generally associated with a parallel increase in antioxidant capacity (Perillo et al., 2020) and thus, the contribution of exercise-induced antioxidant capacity in a malignant context may be controversial. Nevertheless, high levels of endogenous antioxidants from physical exercise may act to protect surrounding noncancer tissue against chemotherapy-induced toxicity (Smuder, 2019).

## Reversible Gene Regulation and Oncogenic Signaling

While genomic material encodes the genotype of an organism, it is the regulation of DNA through epigenetic and transcriptional mechanisms that modulates gene and subsequent protein expression and activity that contribute to phenotype (Mikhed et al., 2015). These reversible modifications can be activated in response to environmental and lifestyle factors (Alegria-Torres et al., 2011) and occur through DNA methylation, histone modification or microRNA expression, with hypermethylation of CpG islands characteristic of both breast and prostate cancer (Garcia-Martinez et al., 2021; Macedo-Silva et al., 2021). More recently, epigenomic approaches have explored how obesity and menopause impact the DNA methylation profile of breast cancer patients, identifying a different epigenome signature in postmenopausal patients with a BMI > 25 compared to premenopausal patients with a BMI < 25 (Crujeiras et al., 2017), suggesting that obesity-induced alterations to the epigenome may contribute to aggressive disease. In addition, hypermethylation of CpG islands through DNA methyltransferase (DNMT) upregulation, inhibits the transcription of various tumor suppressor genes, such as *P21* and *BRCA1*, allowing the proliferation and growth of cancer cells (Banerjee et al., 2014; Pathania et al., 2015). The effects of DNA methylation can be functionally predicted through model-based algorithmic analysis of proteomic data, demonstrating the upregulation of various oncogenic signaling proteins, which then have the potential to further potentiate DNMT hypermethylation *via* a feedback loop system (Emran et al., 2019). While there are several factors that can alter epigenetic mechanisms, ROS have been implicated as a major regulator of transcriptional activity and the cellular proteome (Bhat et al., 2018), and as discussed ROS levels can be regulated through various lifestyle interventions. While the effects of lifestyle choices begin with metabolic changes that influence the epigenome, the subsequently altered epigenome then has the potential to influence the tumor microenvironment. By modifying these lifestyle choices, an array of physiological effects may occur that can impact the risk, progression, and overall prognosis of breast and prostate cancer patients (Figure 2).

## Concluding Remarks

With a global goal of decreasing cancer disease burden, this mini review outlines the physiology behind why lifestyle modifications may succeed as a tool to achieve this. Not only would these changes positively impact the number of cancer diagnoses and outcomes, but it would also concurrently decrease the burden of other worldwide epidemics such as obesity and type II diabetes. While the traditional approach to cancer therapy is dependent on pharmacological interventions, it is now being increasingly recognized that external influence may complement these therapies. These may include, but are not limited to, increasing physical exercise, improving dietary choices, and weight management. Future research should incorporate systems biology techniques to provide a more mechanistic and holistic view on the impact of these modifiable factors on the interactions between the various biological components that contribute to tumorigenesis.

## Author Contributions

KT designed the study, was responsible for writing the article and the creation of all Figures. MN designed the study and was responsible for writing and revising the manuscript. All authors contributed to the generation of the concepts and ideas provided.

## Funding

This work was supported by Cancer Council NSW Research Project Grant (RG 20–08) and Priority-driven Collaborative Cancer Research Scheme (grant #1130499), funded by the National Breast Cancer Foundation Australia with the assistance of Cancer Australia awarded to MN.

## Conflict of Interest

The authors declare that the research was conducted in the absence of any commercial or financial relationships that could be construed as a potential conflict of interest.

## Publisher’s Note

All claims expressed in this article are solely those of the authors and do not necessarily represent those of their affiliated organizations, or those of the publisher, the editors and the reviewers. Any product that may be evaluated in this article, or claim that may be made by its manufacturer, is not guaranteed or endorsed by the publisher.
